# Handlers’ Representations on Therapy Dogs’ Welfare

**DOI:** 10.3390/ani12050580

**Published:** 2022-02-25

**Authors:** Alice Mignot, Karelle de Luca, Véronique Servais, Gérard Leboucher

**Affiliations:** 1 Boehringer Ingelheim, 69800 Saint-Priest, France; karelle.de_luca@boehringer-ingelheim.com; 2 Faculty of Social Sciences, University of Liege, B-400 Liege, Belgium; v.servais@uliege.be; 3 Ethology Cognition Development Laboratory, University of Paris Nanterre, 92001 Nanterre, France; glebouch@parisnanterre.fr

**Keywords:** working dogs, animal assisted interventions, human-dog team, qualitative research

## Abstract

**Simple Summary:**

Most research about Animal-Assisted Interventions (AAI) has focused on the benefits on human health. In contrast, very little has been made on the impact of this work on therapy dogs, although it is part of the ethics of the practice to ensure their welfare. This study aimed to contribute to the knowledge on the welfare of therapy dogs by interviewing 111 handlers through an online questionnaire. The qualitative assessment of handlers’ representations underlined that the welfare of therapy dogs is multidimensional and can be impacted by various variables. Its consideration is important for the quality and safety of the sessions, both for the dog and for the beneficiaries involved. Handlers have a central role in the welfare of their therapy dog and must be trained on stress-related behaviors. Research needs to focus on the impact of interactions on therapy dogs.

**Abstract:**

While research on the benefits of animal-assisted interventions is beginning to build a significant body of work, studies on the well-being of therapy dogs are still in their infancy. Since handlers are the people responsible for their therapy dog’s welfare, we interviewed 111 French handlers through an online questionnaire. Our results underlined that (i) therapy dogs’ welfare is multidimensional when physical and psychological welfare, a balance between work and dog life and the settings and interactions of sessions are all taken into consideration. (ii) The response of our handlers emphasized that considering therapy dog welfare is important for the quality and safety of AAI. (iii) Three categories of risks factors were highlighted: the spatio-temporal framework (planning and environment), the interactions with beneficiaries and the handler themselves. It is particularly important that handlers talk about the negative impact of interactions with beneficiaries since they are at the heart of AAI, however there are few studies focusing on interactions as a stressor for dogs in this practice. Moreover, since there is a potential for positive bias in the handlers’ representations, it is important that they be trained to identify and manage the stress in their dogs. Future research is particularly needed on the impact of interactions during sessions on therapy dog welfare.

## 1. Introduction

During the last 5–10 years, the research interest in assessing the influence of human proximity and/or behavior towards animals on animal welfare has increased [1]. Currently, studies about animal welfare aim to understand the point of view of how animals feel about interactions with humans [2], and this should include working animals. Animal-Assisted Interventions (AAI) are defined as “a goal oriented and structured intervention that intentionally includes or incorporates animals in health, education and human services (e.g., social work) for the purpose of therapeutic gains in humans. It involves people with knowledge of the people and animals involved” [3]. These practices necessarily include an intervening dyad composed of a handler and his/her animal to benefit to a third party (the beneficiary); which is, in fact, different from assistance dogs where the benefits of the dog’s presence are aimed at his/her owner. While the benefits on human health have been well documented (i.e., [4,5,6,7,8,9]), the benefits for the animals are not obvious [10] yet taking into account animal welfare in AAI is ethically crucial and is decisive for the success of the interventions [11]. Indeed, as an interspecies practice, AAI should have an ethos of asking whether the integration of dogs in AAI to promote human wellbeing is at the expense of animal wellbeing? [12]. Interest in therapy animal welfare is not recent, the first alert dates back to 1991 with the study of Iannuzi & Rowan [13], which warned handlers about animal welfare in AAI by focusing on the tiredness and burnout for animals living in institutions. More recently, organizations such as the International Association of Human-Animal Interaction Organizations (IAHAIO) [3] and Animal Assisted Intervention International (AAII) [14] have published guidelines on not only the practices involved in AAI, but also the welfare of the animals involved. Studies about therapy animal (in this paper we use the term *“therapy animal/dog”* because it is the most current term, but it is important to mention that it must change because not all animals in AAI are involved in “*therapy*”) welfare are still in their infancy and concern various species, including dogs (complete reviews in [12,15]), horses (i.e., [16,17,18]) and guinea pigs [19,20]. Focusing on canine species, currently, there is no systematic selection of dogs or required training [21], which can be linked to the variability of contexts in AAI that complicates the application of a selection standard [22]. Indeed, the absence of official regulation in France leads to a heterogeneity of AAI [23,24,25,26] as well as a variety of professionals who unofficially certify dogs. Therapy dog welfare in AAI is a currently concern because interactions with a variety of unfamiliar humans with sometimes unpredictable behavior, (a common occurred in AAI), are potentially stressful for dogs [27,28,29,30]. However, there is currently little consensus regarding the consequences of such interventions on the dogs involved [12] although the integration of animals in AAI is only ethically justifiable if the animal also benefits from the interactions [31]. Moreover, it is important to mention that the physical contact to communicate and tactile interactions are rarely used by dogs [32], although the appreciation of close intimate contact with strangers is an expected behavioral trait in therapy dogs [15,33].

Some research has highlighted differences in salivary cortisol levels between work and non-work days [34,35,36,37] suggesting that working in AAI can stress therapy dogs, while other research has found no differences [38,39,40,41,42,43,44]. On the behavioral observation side, according to the majority of studies, little or no stress behavior is observed in the dogs studied [11,29,39,41,43,44,45,46,47]. This diversity of results could be linked to the diverse spectrum of types of AAIs including a heterogeneity in settings, as well as in the different characteristics of handlers and dogs [10,15,26,48]. Note also that program monitoring and ethical standards related to research effectively limit risks to dog welfare and generalization of results due to lack of fidelity to actual practice [41,49].

Several authors have pointed out that qualitative proxy measures can be reliable in providing a picture of the subjective experience of the animal and thus assess animal welfare [50,51,52,53,54,55], however very few studies have made use of handlers’ perceptions in order to study the manifestation of stress in dogs working within AAI [35,36] despite their responsibility to ensure the welfare of their therapy dog [10]. An additional argument is that general recommendations are unlikely to be sufficiently acted upon if the handlers do not see the need for them or if they are not adapted to their working conditions. Consequently, in this study, we chose to place the representations of handlers regarding their therapy dog’s welfare under the spotlight in order to identify what stands out the most, and to pinpoint their biggest concerns to develop a basis for future studies and recommendations.

Objectives: This exploratory study interviewed handlers in order to understand how they perceive their therapy dog’s welfare so as to highlight the importance of addressing the well-being of therapy dogs as well as to indicate the environmental and interactional risk factors to this well-being.

## 2. Materials and Methods

### 2.1. Sample and Recruitment

This study is included in a doctoral project investigating the French practice of AAI. We questioned French handlers in AAI who agreed to answer to an online questionnaire shared on AAI-specialized social media accounts and sent by emails from April 2018 to May 2019. Our inclusion and exclusion criteria were very limited since we wanted to have a real vision of the field. We aimed to include workers from different fields of animal mediation (recreational, therapeutic, social, etc.). We have therefore included all handlers currently working in AAI with at least one dog and wishing to participate in our research on a voluntary basis, regardless of their professional background or affiliation to AAI associations. We focused mainly on canine-assisted interventions as the dog is the most common animal species in AAI [56,57,58,59]. The questionnaire covered several aspects of working with dogs in a therapeutic setting; this article focuses on identifying risks to the welfare of therapy dogs, based on an analysis of handlers’ representations in this regard. See Mignot [18] for the representations of French handlers on their AAI practices and Mignot [26] for the characteristics of the French practice of AAI in relation to the IAHAIO model.

#### Ethics

The Ethics Committee of the UFR SPSE (Psychological and Educational Sciences University Paris Nanterre) approved this investigation, protocol code is 04-n°5. Before accessing the questionnaire, handlers were required to complete a consent form that included an explanation of the study framework, objectives and the research ethics features.

Signing this consent form guarantees the confidentiality of their responses, the possibility of interrupting the research, respect for their integrity and their rights in accordance with the research ethics. The collection, processing and storage of personal data complied with the rules laid down by the General Data Protection Regulation [60].

### 2.2. Data Collection

A five-section questionnaire was constructed based on a literature review [3,23,36,61,62,63,64,65] and questioning raised during informal interviews and internship. As mentioned before, for this article, we focused our attention on 10 items (Table 1; complete questionnaire in Mignot [48]).

The first items concerned general questions about handlers’ representations on therapy dog welfare: the definition of dog welfare in AAI, its impact on the quality of a session, the handlers’ application of the main guidelines concerning therapy dog welfare, the limitations encountered to respect therapy dog welfare and their representations about the benefits for therapy dogs to work in AAI. The second part concerned the risk factors identified that could affect therapy dog welfare: the parameters influencing therapy dog welfare, the factors that stress their dogs, what handlers put in place to ensure the welfare of their therapy dog and also how they observe stress and pleasure in their dogs.

### 2.3. Analyses

A descriptive analysis was performed by calculating means and frequencies for numerous and categorical variables with the software GraphPad Prism 9^TM^. We chose to categorize the answers to the open questions into themes: the parameters affecting therapy dog welfare, the risks factors identified, and the way handlers observed stress or pleasure in their therapy dogs.

A qualitative descriptive method was selected for the five open questions: the definition of dog welfare in AAI, the importance of therapy dog welfare for a good session, the benefits for the dogs working in AAI, what handlers put in place for their dog’s welfare and the limitations encountered that may prevent the recommendations from being respected. We used a phenomenological method because it “describes the meaning for several individuals of their lived experiences of a concept or a phenomenon; describing what all participants have in common as they experience a phenomenon” [66]. Like Firmin et al. [61], we used an open coding strategy with a line-by-line analysis approach to explore data for recurring ideas [66,67,68], and developed clusters of meaning into themes. Only the principal investigator conducted the qualitative analysis and did it twice 3 months apart to confirm that the same themes emerged from the data. Also, the relevance of the themes and the mapping that emerged from the initial coding were discussed with the other researchers. We then counted the number of themes mentioned by each handler (a response could concern several themes) and finally calculated the percentages of each theme on the total number of themes cited for each question. These analyses respect the 15-point checklist of criteria for thematic analysis underlined by Braun and Clark [69]. Since it is a study that aims to show what stands out the most in the handlers’ representations, we presented the major themes for each question, all themes are presented in the tables.

## 3. Results

### 3.1. General Characteristics of the Human-Animal Team

111 French handlers in AAI completed the entire questionnaire. The complete characteristics of our sample are presented in our precedent articles [26,48]. Regarding handers, our sample was composed of 94.59% (*n* = 105) female and 5.41% male (*n* = 6). Their mean age was 41.28 years with a minimum of 20 years and a maximum of 68 years. 83.78% (*n* = 93) of handlers had training in AAI, mainly in private training centers (80.65%; *n* = 75). They were primarily trained in AAI by various private small centers (36.56%; *n* = 35). Handlers initially trained in the animal field represented 37.84% (*n* = 42) of our sample. They were mostly represented by dog trainers (50%; *n* = 21), veterinarians/assistants (19.05%; *n* = 8), training in handling visiting dogs (14.29%; *n* = 6), training in handling assistance dogs (7.14%; *n* = 3), breeders (7.14%; *n* = 3), and an ethologist (2.38%; *n* = 1).

Handlers were able to answer for one or two therapy dogs. As a result, 57 handlers responded for only one dog and 54 for two dogs. From a total of 165 dogs, 63.63% were females (*n* = 93) of which 72.04% were sterilized (*n* = 67); 58.33% of males were neutered (*n* = 42). Their mean age was 5 years (±0.26) and they began AAI at 24.02 months (±2.05). Breeds were mostly retrievers (32.92%; *n* = 53), with only golden retrievers and labrador retrievers, shepherd dogs (24.22%; *n* = 39) with mostly Australian shepherd, Shetland shepherds and border collier and companion and toy dogs (13.66%; *n* = 22) with mainly bulldogs, cavalier king charles spaniels and poodle. Almost half of the dogs were certified (44.85%; *n* = 74), and this was mostly through 11 different private AAI associations (35.14%; *n* = 26).

### 3.2. Handlers’ Representations of Therapy Dog Welfare

In this section, we grouped handlers’ answers to open questions regarding their definition of therapy dog welfare, how a dog’s welfare can impact a “good session” and the benefits of working in AAI for their dogs. All themes are presented in the tables.

#### 3.2.1. Definition of Dog Welfare in AAI 

For the question about their definition of therapy dog welfare, handlers mentioned one to five themes in their answers. We identified seven major themes for the definition of dog welfare in AAI (Table 2). The three major themes most cited by handlers were the psychological welfare of dogs, followed by the respect of the needs and rhythm of the therapy dog, and the consideration of therapy dog as an individual.

#### 3.2.2. Impact of Therapy Dog Welfare on a Good Session of AAI 

Regarding the impact of dog welfare on a “good session”, 108 handlers answered “*yes*” to this question and then answered the open question regarding the reasons they thought this. 97 handlers answered to this question and mentioned one to four themes in their answers. We identified eight major themes for the impact of dog’s welfare on the session of AAI (Table 3). The three major themes most cited by handlers were the quality of interactions, followed by the attentiveness of the dog during session and the emotion contagion.

#### 3.2.3. Benefits for the Dogs to Work in AAI 

For the question about the benefits of working in AAI for dogs, handlers mentioned one to five themes in their answers. We identified seven major themes for the benefits for the dogs to work in AAI (Table 4). The three themes most cited by handlers were the positive interactions with humans, followed by being with their human and the cognitive and physical stimulations.

### 3.3. Parameters Influencing Therapy Dog Welfare

In this section, we grouped questions on what handlers put in place to ensure their therapy dog welfare, their application of major recommendations and the limitations encountered when attempting to apply these recommendations.

#### 3.3.1. Handlers’ Actions to Respect Therapy Dog Welfare 

For the question about handlers’ actions to respect their therapy dog welfare, handlers mentioned one to six themes in their answers. We identified eight themes in their answers (Table 5). The three major themes cited by handlers were the times to unwind, their active role during session and the management of environmental equipment.

#### 3.3.2. Recommendations 

Regarding handlers’ application of the main recommendations concerning therapy dog welfare, (Table 6), the highest mean scores were for the cessation of sessions when the dog is stressed, the recognition of stress signals, the use of positive training method, while the lowest mean scores were for “time-outs” during sessions, the time for adaptation before sessions and the cessation of sessions before appearance of stress signals.

#### 3.3.3. Limitations to the Application of These Recommendations 

After answering the precedent question, handlers were able to elaborate on the limitations they encountered during the practice with regards to respecting the precedent recommendations. Handlers mentioned one to three themes. We identified seven major themes for the limitations to the application of major recommendations (Table 7). The three major themes most cited by handlers were the environment, followed by the organization and the expectations of the institutions.

### 3.4. Risk Factors for Dog’s Stress 

The interrogation about the risk factors identified by handlers for therapy dogs’ welfare was divided into two questions: “*According to you, what are the three parameters that most influence the well-being of the dog in AAI?*” and “*With regard to each dog, what do you think stresses him/her?*” We categorized all factors into 14 categories (see Table 8) and grouped both answers into one analysis.

**Figure 1:**
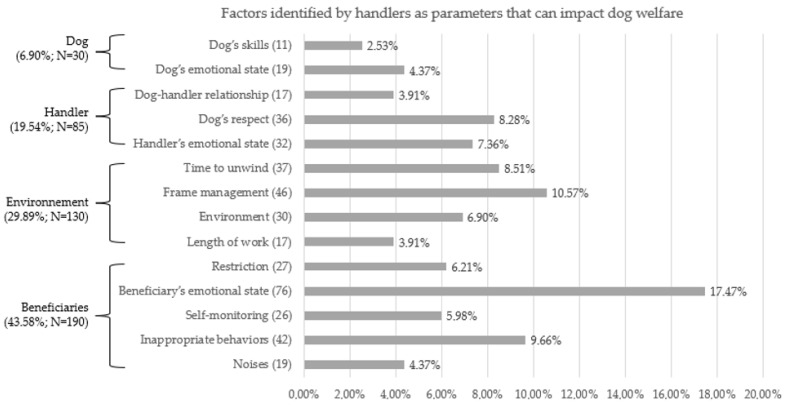
This figure represents the factors identified by 111 handlers as parameters that can impact therapy dog welfare. *n* = 435 themes categorized in 14 themes. Values are indicated next to the theme in brackets.

For the questions about the risks factors for dogs’ stress and welfare in AAI, we analyzed 435 factors (Figure 1). The factors most cited were the beneficiary’s emotional state, followed by the frame management and inappropriate behaviors. By grouping our data into general categories, we underlined four major factors: the beneficiaries, the environment, the handler (19.54%; *n* = 85) and the dog (6.90%; *n* = 30).

### 3.5. Communication of Dogs

#### 3.5.1. Dog’s Communication of His/Her Limits

For the question about the way handlers perceived that their dogs communicate their limits, we coded the answers into six categories (Table 9). Handlers mostly noticed the limits of their dogs by the fact that their dog goes away, followed by the calming signals and the avoidance of interactions with beneficiaries. Handlers also mentioned the contact seeking with them that can be physical or visual. Finally, handlers mentioned the decreased in attention (7.02%; *n* = 12) and the agitation (5.26%; *n* = 9).

#### 3.5.2. Signs Showing Pleasure in Dogs

For the question about how handlers perceive pleasure in their dogs in AAI, we coded 168 terms. Five major themes were identified in handlers’ answers (Table 10). The search for interactions was the most represented, followed by the pleasure signals, which included cheerfulness, tail wagging, a playful attitude, jumping and barking. Handlers also noticed the motivation to go to work with the excitement at seeing work -related paraphernalia or running to the care facility etc. Finally, they mentioned active participation and a relaxed attitude.

## 4. Discussion

The aim of this study was to interrogate handlers in order to understand how they grasp the welfare of their therapy dog (i) to highlight the importance of addressing the well-being of therapy dogs both for dogs and beneficiaries as well as (ii) to point out the environmental and interactional risk factors to this well-being. To this end, we considered handlers’ representations as it may allow the identification of risk factors for the well-being of therapy dogs in complementarity to the results of studies using physiological indicators and/or behavioral assessment.

We assume that our sample is representative of the French practice of AAI because handlers were dispatched in various localizations in France. In addition, as there is no official data about the number of handlers who practice AAI in France, this study can be considered as a pilot for further investigations. Our sample represented a variety of French practices in AAI regarding the characteristics of the human-dog teams. Indeed, handlers were mostly trained in AAI whereas it is not mandatory in France [23,26,70]. It is important to note that they were trained in different structures, which can impact their representations and management of their therapy dog welfare [48]. Only 37.84% of them undertaken some initial training within the animal field, which was represented by various professions (i.e., veterinarian and breeder). Therapy dogs also represented various profiles regarding their gender, age, starting age in AAI and breeds. Beyond the heterogeneity of human-dog teams that corresponds with the heterogeneity of AAI in France, this highlights that any action for the well-being of therapy dogs must target the different characteristics of handler and dogs involved in AAI.

Since we questioned the welfare of therapy dogs in different ways, we chose to present handlers’ general representations on the definition of therapy dog welfare first, then their representations about the importance of considering the welfare of therapy dogs, and finally the risk factors identified by them coupled with how they identify stress in their dogs. Once again, our objective was to show what stands out most in their representations in order to find areas for study and intervention.

### 4.1. Generalities on Dog Welfare in AAI

Answers about the definition of therapy dog welfare varied from one handler to another, but their answers converged on the importance of a balance between worktime and the dog’s life, as well as their psychological and physical welfare and the considerations of therapy dogs as individuals with specific needs and preferences.

The first theme cited by handlers concerned the psychological welfare of therapy dogs (32.37%) with the absence of negative emotions (including mostly stress) and the presence of positive emotions during sessions. It corresponds to the definition of animal welfare that included the absence of stress [71,72,73,74] and the presence of positive emotions; that has been included more recently in the assessment of animal welfare [11,75,76]. On a smaller percentage, handlers cited the “physical” welfare of therapy dogs (7.69%) that included mostly the absence of injury during sessions but also the respect of the specific needs of the canine species. Some handlers mentioned, on the borderline of psychological and physical well-being, that it is important for therapy dogs to participate without coercion either through coercive tools or mental restraints [11]. To guarantee the absence of stress during sessions, it is important for handlers to be able to recognize stress signals in dogs. Indeed, the active role of the handler was the fourth theme (12.56%) cited by handlers, notably with noticing stress signals and removing the dog from interactions. However, this can be difficult when handlers are not trained and may also be biased because they want their dog to enjoy accompanying them to work. This positive bias has been highlighted in the article of Zenithson [10] and confirmed in the study of Haubenhofer and Kirchengast [35] where handlers used positive words (satisfied, relaxed, happy mood) to describe how their dogs feel after a AAI session.

The second theme mentioned by handlers in their definition of their dog’s welfare in AAI was the fact that the dog should have a dog’s life outside of work (21.74%). This theme mostly regrouped time away from work to unwind, blow off steam or rest. It underlines that, for the handlers, it is important that their dog also has a “dog’s life” outside of the work and that the needs of the canine species are taken care of (walking, playing, meeting fellow canines). Developing upon these thoughts, the evocation of the importance of a balance between work and time-off to unwind suggests that handlers consider that working in AAI can be stressful for their dogs and that they must decompress. Indeed, some studies [34,35,36,77] have underlined a higher level of stress on work days than on a day “off”; supporting handlers’ perceptions.

The third theme mentioned by handlers was to consider their dogs as individuals who have their own limits and preferences and the ability make choices (14.78%). This means that what suits one dog will not necessarily suit another and that an adaptation of the working conditions is necessary. This individuality must also be considered in relation to the characteristics of the setting and all the variables with which the dog must cope [22]. Furthermore, they evoked the freedom of their therapy dog that included not only the possibility for them to make choices such as whether or not to come to the sessions and participate but also in the management of the space during sessions (i.e., having a place to rest). Therefore, handlers also evoked a mutual comprehension with their dogs and the necessary adjustment during sessions based on their dog’s signals.

Finally, since the goal of this study was to get a complete picture of the welfare of therapy dogs, we also asked handlers about the perceived benefits of AAI for their dogs. It seems that the handlers see the work in AAI as something positive for their dogs because it allows them to not be alone at home, to experience positive interactions with other humans, to become familiar with different environments and to have their cognitive needs met. However, it is important to note that it is normal for handlers to see benefits for their dogs, but this has to be put in perspective with each dog’s personality and the way the sessions are conducted. For instance, handlers mentioned being in the presence of their humans and/or other humans as a primary benefit for therapy dogs, which could be discussed because interactions between human and dogs could be positives for both [78,79] but also stressful [27,28,29,43,80].Handlers also cited the physical and cognitive benefits for therapy dogs with training and stimulation before and during sessions. Indeed, the behavioral training for AAI provide positive mental stimulation [31]. Consequently, some handlers made the comparison with the classic “pet dog” that stays alone all day, which can indeed affect such a dog’s welfare [81].

To summarize, handlers’ answers underline the fact that dog welfare in AAI involves common prerequisites for all therapy dogs (time-off, psychological and physical integrity) but also specificities linked to each dog. Consequently, it seems important to consider the balance between the costs and benefits of each session for each dog.

### 4.2. Importance to Take into Account Dog’s Welfare in AAI 

The interviews with the handlers about how therapy dog welfare can affect a session of AAI underlined three main points: the influence of therapy dog welfare on the quality of work in AAI, the risks for beneficiaries of interacting with a stressed therapy dog and the risks for the therapy dog him/herself. The first point mentioned by handlers concerned the influence of therapy dog welfare on the quality of the session, which was mostly represented by the quality of interactions, the attentiveness and the motivation of the dog In the handlers’ discourses, we found the general idea that a dog in discomfort will be focused on managing his stress rather than on meeting the beneficiaries and listening to their requests. Consequently, they evoked the avoidance of interactions as a sign that their dog is in discomfort, and the search for interactions and the motivation to go to work as a sign of pleasure. This links with the idea that the well-being of the therapy dogs plays on the quality of their work. Indeed, the main expectation on therapy dogs is to initiate interaction and bonding with the beneficiaries [12,82,83,84]. Handlers also mentioned the possible impact of negative therapy dog welfare on the safety of the beneficiaries with the risk of agitation or biting in a stressed dog. Indeed, a depiction of the gestures that any dog will give in response to an escalation of perceived stress and threat has been illustrated by the Canine ladder of aggression of Sheperd [85]; from yawning, blinking, and nose licking to biting. In the behaviors identified by handlers as a communication of limits, handlers mentioned appeasement behaviors that have been identified as signs of stress in the literature [86,87] and are at the lower end of the aggression scale. These behaviors must therefore be perceived by handlers and considered to guaranty the safety of beneficiaries. Some handlers also mentioned that the stress of dogs can be “contagious” for beneficiaries if they feel the dog’s discomfort. Finally, handlers mentioned the importance of considering a therapy dog’s welfare because due to the risks for the dogs in relation to the ethics in AAI and a parallel with the burnout of caregivers after chronic stress. It is interesting that it was not the prior theme mentioned by handlers about the impact of dog welfare on a good session, evoking that a session can be good even if the dog is in poor welfare.

### 4.3. Risks Factors

We grouped what handlers cited as stress factors and/or parameters influencing therapy dog welfare into four categories: the interactions with beneficiaries, the space-time framework, the handler’s responsibility, and the dogs’ characteristics. We discuss only the first three because as we don’t have sufficient details regarding the characteristics of dogs as risks factors for their welfare, the dog’s emotional state during sessions and the dog’s skills more generally.

#### 4.3.1. Interactions

The influence of interactions represented 43.68% of identified stress factors by handlers. As mentioned before, interactions between human and animals could be a benefit for dogs, but it can also be stressful [27,28,29,43,80]. Interrogated handlers evoked three types of human behaviors that can affect therapy dog’s welfare Firstly, handlers evoked human behaviors that are not directly threatening to the therapy dogs but can be stressful such as restrictive behaviors and behaviors that require the dog to control him/herself. Indeed, being approached, petted on the head, kissed or hugged by strangers can play a role in the comfort level of therapy dogs [12,88], whereas these situations are common in AAI [89]. Secondly, handlers mentioned the presence of behaviors that can be perceived as stressful and/or painful by therapy dogs such as agitated or even aggressive behaviors, which could be linked to the beneficiaries’ pathologies. Even if these behaviors are not directly directed toward the dog, they can be interpreted as a threat by the dog. Finally, handlers mentioned inappropriate behaviors directed towards the therapy dog such as abrupt gestures, rough handling and direct interactions. The mistreatment of therapy dogs is rarely mentioned in studies [12] or by handlers [10] although it is a reality in the field. As Beck & Katcher [90] point out, research needs to identify populations or situations where contact with therapy animals may be problematic or inappropriate for both the animals and the people involved. Therefore, this may call into question the idea of handlers that working in AAI represents a social benefit for therapy dogs. The positive or negative impact of the beneficiary-dog interactions must then be put into perspective with the type of interactions that take place in the session. In addition, in our study the presence of a referent responsible for the beneficiaries in the AAI was random, which puts the handler in the position of being the only one responsible for these interactions. It is important that handlers work in collaboration with a referent that regulates beneficiary behaviors and who is also trained to recognize a dog’s stress.

#### 4.3.2. Space-Time Framework

The space-time framework represented 28.89% of risks factors cited by handlers. They mentioned the length of work (frequency and duration of sessions) and the environment (size of the room, number of beneficiaries) as important parameters that influence therapy dog welfare. It is congruent with the literature that mentioned an effect of the duration of sessions [13,34,36], the frequency of sessions [13,34,40,41] and the number of beneficiaries [40] on a therapy dog’s welfare. We can also include in the framework of AAI the mention of a balance between work and time-off that should be considered to be part of the dog’s work schedule. Finally, the medical staff and the institutions were pointed out by handlers as a limit for dog welfare. Indeed, they evoked the difficulty of respecting the needs and choices of the dog the expectations and organization of institutions. This underlines the fact that these institutions do not always seem to understand that AAI is a practice with living beings and that expecting a certain “profitability” from the animal is inappropriate. Indeed, some authors mentioned that the economic interests could lead to a conflict of interest if they outweigh the welfare status of the animal [10,12]. It is therefore important that institutions understand the importance of considering therapy dog welfare and that there is no pressure to be cost-effective with AAI. On the other hand, handlers must be trained to be able to meet the expected objectives, even without the active presence of their animal.

#### 4.3.3. Handlers’ Responsibility

Handlers considered themselves as an important factor (19.54%) that can affect their dogs’ welfare because they are the ultimate gatekeeper of animal welfare in AAI [58]. Indeed, all factors mentioned above are under the responsibility of handlers (except the influence of the institution in which they work). Therefore, they evoked a role in prevention by constructing a framework that respects the dog’s needs, their preferences and limits; and by supervising the interactions with beneficiaries during sessions. For the framework, the handler is not only the responsible of the planning but also for the management of the space. For instance, handlers can organize the space of the session with a safe place for their dogs to escape from interactions if they are stressed or tired [15,91]. As mentioned before, handlers must adjust to their dogs, not force interactions with the beneficiaries and leave a margin of control to their dogs. During interactions, handlers have a role of mediator between their dog and the beneficiaries and must intervene in the early stages of negative arousal [12] and give breaks before discomfort appears [10]. In our “recommendations” section, the highest mean score was for recognizing signals of stress and subsequently stopping sessions when the dog is already stressed, but a low mean score for stopping sessions before the stress appears. This can be due to the variation of knowledge on animal behavior between handlers that can impact their perceptions [91]. As we mentioned before, handlers having training in AAI from various structures leads to uneven knowledge on canine behavior and welfare. Moreover, the perception of stress in their dogs can vary between humans [92], especially when speaking about subtle signals of stress. In our sample, handlers underlined four types of signals indicating stress in their dogs: the avoidance of interactions, appeasement signals, agitation and physical or visual contact seeking. It underlined the variety of signs of stress in dogs, which is congruent with the literature [93,94]. Finally, regarding their relationships, handlers mentioned the influence of their own emotional state on their dog’s stress. It has been mentioned recently in a paper that handlers may suffer from compassion fatigue, which can compromise their own welfare but also the ability to lead AAI [95].

Therefore, a lack of training in dog behavior can be a risk factor for a therapy dog’s welfare; they therefore must be accompanied by professionals such as dog trainers or specialized ethologists.

## 5. Limitations

The representativeness of our sample could be questioned because the handlers who chose to answer to our questionnaire were concerned by their practice in AAI and the selection of their mediation dog. In addition, we chose to study the representations of the handlers as an approach to understanding the well-being of the therapy dogs, however it is possible that their answers are biased by a desire that the work pleases their animal as well as a social desirability bias. By looking at the social desirability bias, we can think that, since the questionnaire was entirely anonymous, the handlers did their best to transcribe the reality of their AAI practices. The combination of interviewing handlers and directly observing their practices would have countered these biases. Therefore, these data should not be understood as truth but as possibilities for exploration for future research. Future research should focus on behavioral analyses of various animal-mediated devices with a focus on interspecific interactions and their potential for stressing dogs.

## 6. Conclusions

The interview of handlers about therapy dogs welfare underlined their concerns about their dogs’ well-being. They have ideas about the factors that can positively or negatively affect well-being and they adjust their practices according to these ideas. Handlers’ representations about therapy dogs welfare highlight the need to consider it as multidimensional with all the variables it encompasses such as the spatio-temporal framework (planning and working area), the individuality of each dog, the beneficiaries and interactions, and the handler’s own knowledge and skills. This underlines the importance of thinking about therapy dog welfare within a context that encompasses all of the variables that can affect it. Furthermore, handlers’ answers emphasized the importance to integrate the concept of one welfare in the considerations and studies about AAI [96,97,98]. Developing upon these thoughts, it is important to note that there may be a bias in the handlers’ representations related to the fact that they want their dog to enjoy working with them. This bias must be considered particularly in relation to the interactions with beneficiaries but also the choice of the setting in which the dog works. It is important that the handler is trained to recognize the signs of stress in their dog and not leave them in an uncomfortable situation. As handler’s are the principal person responsible for their dog welfare it is important to make sure that they are trained in dog behavior and that the institutions give them full control over the management of the framework based on their expertise of their dog. Research in applied ethology must be developed to provide science-based criteria to assess therapy dog welfare [62,99] to give clear guidelines to handlers and, on the other side, as experts of their dogs handlers’ voices must be legitimize in research about AAI.

## Figures and Tables

**Table 1:** Description of the section “dog’s welfare” of the questionnaire used in this study. Complete description of the questionnaire as a supplement data in Mignot [48].

Questions	Type
Generalities on therapy dog welfare	
How would you define dog welfare in AAI?	Open question
Do you think that the dog’s well-being plays on a good session? If so, why?	YES/NO Open question
The following is a cross-reference of the main guidelines found in the studies/associations. Indicate for each one whether you apply it: never, rarely, sometimes, often, all the time.	5-points Likert scale
What limitations do you encounter that prevent you from following these recommendations?	Open question
What are the benefits of AAI for your dog?	Open question
Risks factors identified that could affect therapy dog welfare	
In your opinion, what are the 3 parameters that have the most influence on it?	Open question
According to each dog, what do you think is stressing him?	Open question
What do you put in place to respect your dog’s well-being?	Open question
How does your dog communicate his limits to you during a session?	Open question
What are the signs that your dog is enjoying AAI?	Open question

**Table 2:** This table represents the answers to the question “How would you define dog welfare in AAI?”. *n* = 101 complete answers. Total of 207 terms coded grouped into 7 themes; presented in descending order.

Themes	Description	*n*	%
Psychological welfare	Presence of positive emotions, relaxed attitude, pleasure; absence of stress, fear, tiredness and negative emotions	43	32.37%
Dog’s Needs/rhythm	Dog’s life, walk, play, “off life”; rest time, unwind, not too much work	45	21.74%
Dog as an individual	Dog with his own preferences and limits, the respect of his choices (coming to the session, participating in an activity/interaction)	31	14.98%
Active role of handler	Noticing stress signals, remove the dog from interactions	26	12.56%
Motivation	Willingness to come and to participate, seek interactions, dynamic, availability, listening	21	10.14%
Physical welfare	Absence of injury, respect the specific needs of the species, resting place, water	17	8.21%

**Table 3:** This table represents the answers to the question “Do you think that the dog’s well-being plays on a “good” session? If so, why?”. *n* = 97 complete answers. Total of 133 terms coded grouped into 8 themes; presented in descending order.

Themes	Description	*n*	%
Quality of interactions	Less initiation and reception or even reluctance for interactions with beneficiaries, “not a good mediator”	37	27.82%
Attentiveness	Not focused on the session; unable to think; not listening	19	14.29%
Emotional contagion	“A happy animal shines”; unhappiness is perceived by the beneficiaries; if the dog is not relaxed, neither is the handler;	15	11.28%
Quality of work	Cannot work properly, brings nothing to the session; it’s not therapeutic	11	8.27%
Motivation	Willingness to intervene, waiting for next session	14	10.53%
Ethic	There can be no aai without dog welfare	13	9.77%
Risks for the dog	Parallel with humans; not to exhaust him	12	9.02%
Safety of beneficiaries	Biting, agitation, inappropriate behaviors	12	9.02%

**Table 4:** This table represents the answers to the question “What are the benefits of working in AAI for your dog(s)”. *n* = 101 complete answers. Total of 201 terms coded grouped into 7 themes; presented in descending order.

Themes	Description	*n*	%
Positive interactions with humans	Sharing positive moments with humans, being petted, be the center of attention, need for human contact	71	35.32%
Being with their humans	Pleasing their human, working with their human, sharing time with their human, enhancing the relationship	50	24.88%
Cognitive and physical	Being in action, being stimulated, mental stimulation	21	10.45%
Play	Playing, doing activities	19	9.45%
Treats	Treats	14	6.97%
Socialization	Discovering new places; learning to manage new situations; increasing adaptability	13	6.47%
Don’t stay alone at home		13	6.47%

**Table 5:** This table represents the answers to the question “What do you put in place to respect your dog’s well-being?”. *n* = 109 complete answers. Total of 262 terms coded grouped into 8 themes; presented in descending order.

Themes	Description	*n*	%
Times to unwind	Walks to let off steam or to relieve oneself; before and/or after a session; regular walks; scheduled or as-needed breaks; rest/relaxation time; play	55	20.99%
Handler’s active role	Stop interaction; put the dog away; know their animal; check its condition; be alert to stress signals; be vigilant	36	13.74%
Environmental equipment	Resting place; water	34	12.98%
Adjustment	Choice of activities according to the dog’s condition and preferences; change of activity if necessary;	26	9.92%
Implication of beneficiaries	Explanations of the dog’s behavior; reminders of the rules to be respected; verbalizing the emotions	21	8.02%
Organization	Duration and frequency of sessions; number and type of beneficiaries; session rituals; schedule management	21	8.02%
Freedom	Ability to withdraw; lack of coercion; choice in interactions	20	7.63%
Treats	As a reward or to keep him busy	11	4.20%

**Table 6:** This table represents the answers to the question “The following is a cross-reference of the main guidelines found in the studies/associations. Indicate for each one whether you apply it never, rarely, sometimes, often, all the time »; *n* = 111 complete answers.

Recommendations	Mean	SEM
Cessation of session when the dog is stress	4.818	0.05195
Recognition of stress signals	4.564	0.05407
Positive training method	4.564	0.0817
Choice to interact	4.555	0.06659
Assessment of physical and behavioral states	4.527	0.08240
Possibility to unwind before and after session	4.440	0.08882
Presence of a rest place	4.382	0.09312
No leash during sessions	4.239	0.08831
Choice to come and to participate	4.145	0.1007
Cessation of session before the apparition of stress signals	3.855	0.09209
Time for adaptation before session	3.736	0.1223
Time-out during session	3.318	0.1323

**Table 7:** This table represents the answers to the question “What limitations do you encounter that prevent you from following these recommendations?”. *n* = 57 complete answers. Total of 72 terms coded grouped into 7 themes; presented in descending order.

Themes	Description	*n*	%
Environment	Small rooms; inability to leave the room; environment; open room	22	30.56%
Organization of work	Sequence of sessions; difficulty in taking a break; pressure to be on time; no warm-up time; contingencies	19	26.39%
Expectations of the institutions	Difficulty in accepting that the caregiver comes without his dog if the latter is sick; the desire to keep a contract with the institution even if the latter does not seem to be interested in the dog’s well-being	10	13.89%
Beneficiaries	Types of patients (ages, pathologies); inappropriate behaviors	9	12.50%
Presence of leash to control the dog	Presence of cats; food; rough dog requiring a leash	5	6.94%
Selection of the therapy dog	No external evaluation	4	5.56%
Handler	Fatigue; alertness; difficulty recognizing stress signals	3	4.17%

**Table 8:** Description of the categories selected for the analysis of risks factors for therapy dog welfare.

Categories	Description
**Interactions**	
**Self-monitoring**	Management of the dog’s frustration that it is linked to poorly formulated requests, inappropriate gestures, etc.
**Noises**	Shouting of the beneficiaries, incomprehensible noises
**Type of interactions**	Abrupt/inappropriate gestures; positive vs. negative interactions
**Restriction**	It can be unwanted/unappreciated physical contact, intrusion into personal space, but also being surrounded by too many people. This notion thus includes the non-respect of one’s limits, everything that is an obligation to interact but also to work.Not obliged to, not forced, freedom, being surrounded, hugs too tight, being on the knees
**Beneficiary’s emotional state**	The mood of the group, agitation, restlessness, anger directed or not directed at the dog, excitability; also symptoms of pathology
**Work environment**	
**Length of work**	Duration of the sessions and their frequency
**Time to unwind**	rest time; walks
**Environment**	Everything related to the physical space where the animal mediation takes place: Size of the room, resting area etc., novelty
**Framework management**	Management of the framework by the handler: both the physical framework and the interactions, also the proposed activities; framework imposed on the beneficiaries and factors related to the organization: number of participants number of requests, external solicitations, protection in case of discomfort, framework imposed on the beneficiaries, temperature, organization of the institution
**Handler**	
**Handler’s emotional state**	My mood, stability, nervousness, not being self-confident
**Dog-handler relationship**	Everything that is positive between them: trusting relationship but also feeling secure and listening to the dog, reading the dog, attention but also knowledge; Confidence in one’s human being, feeling of security
**Dog’s respect**	Respect for the dog/his needs/listening to the dog = the attention given to his needs and behavior during the session; Attention to the dog, respect for its needs, ability to understand signals, knowledge of canine behavior, limits.
**Dog**	
**Dog’s emotional state**	Mental/mood of the day/state of health—also general wellbeing; Dog available, in good health; Fatigue
**Dog’s characteristics/skills**	Basic characteristics of the dog that may or may not allow him to manage his emotions; Socialization, fear, selection of the dog, education of the dog

**Table 9:** The table represents handlers’ representations on how their dog communicate his limits during sessions; *n* = 171 terms coded.

Categories	Total (*n* = 171)	%
Dog goes away	53	30.99%
Appeasement signals	31	18.13%
Dog stays but avoid interactions	25	14.62%
Visual contact seeking	23	13.45%
Physical contact seeking	18	10.53%
Decreased attention	12	7.02%
Agitation	9	5.26%

**Table 10:** This table represents handlers’ answers on how their dog communicates his pleasure during sessions; *n* = 168 terms coded.

Categories	Total	%
Seeking for interactions	64	38.10%
Pleasure signals	47	27.98%
Motivation to go to work	33	19.64%
Active participation	15	8.93%
Relaxed attitude	9	5.36%

## Data Availability

Not applicable.
